# Voluntary Dissociation of Motor Unit Activity in the Vastii Muscles

**DOI:** 10.1523/JNEUROSCI.1982-25.2026

**Published:** 2026-04-24

**Authors:** Daniel Haller, Finja Beermann, Raul C. Sîmpetru, Luca Hofbeck, Roger M. Enoka, Alessandro Del Vecchio

**Affiliations:** ^1^Neuromuscular Physiology and Neural Interfacing Laboratory, Friedrich-Alexander-Universität Erlangen-Nürnberg, Erlangen 91052, Germany; ^2^Traumatology and Orthopaedics, Universitätsklinikum Erlangen, Erlangen 91054, Germany; ^3^ Department of Integrative Physiology, University of Colorado Boulder, Boulder, Colorado 80309-0354

**Keywords:** intramuscular EMG, motor unit recruitment, muscle synergies, vastus lateralis, vastus medialis, volitional motor control

## Abstract

The central nervous system controls movement with consistent activation patterns across muscles and motor units (MUs), suggesting the presence of a relatively fixed and high-dimensional number of neural constraints on voluntary actions. In the human quadriceps, the vastus medialis (VM) and vastus lateralis (VL) contribute to the knee extensor torque and are considered a synergistic pair largely activated by shared neural inputs. However, some evidence suggests that these muscles or even subregions within them can be controlled independently. We investigated whether humans can dissociate neural input to VM and VL during isometric contractions. Ten participants (six males, four females) received real-time feedback from multiple intramuscular electromyography (EMG) electrodes inserted into different regions of the VM and VL while attempting to activate each muscle or region selectively. Nine out of ten participants were able to separate VM and VL activity based on the intramuscular EMG feedback. However, MU decomposition from the intramuscular EMGs revealed that selective recruitment of a unique set of MUs was possible only within the proximal region of VM. In contrast, we found highly correlated activity between MUs in VL and distal VM. Correlation analyses confirmed that the proximal VM exhibited distinct activation profiles compared with both distal VM and VL, supporting the existence of compartmentalized control within VM. These findings demonstrate that it is possible to dissociate the activation of MUs within this synergistic muscle group during low-force isometric contractions.

## Significance Statement

Humans are typically thought to lack voluntary control over individual quadriceps muscles due to a shared neural input and a common distal tendon. With real-time EMG feedback from multiple muscle implants, we found that participants were able to activate distinct motor unit populations within vastus medialis, partially dissociating its activity from the vastus lateralis. These results reveal a relatively flexible, region-specific neural control within a pair of synergistic muscles that offers new perspectives for motor learning and targeted rehabilitation.

## Introduction

The central nervous system (CNS) simplifies movement control by distributing common synaptic inputs to groups of motor neurons, forming functional units often referred to as muscle synergies or neural modules. This framework proposes that motor behavior is generated by projecting a limited number of neural commands onto sets of motor units (MUs) within and across muscles (de Luca and Erim, 1994; Ivanenko et al., 2004; d’Avella et al., 2006; Laine et al., 2015; Negro et al., 2016b).

The prevailing view within the quadriceps muscle group has long been that the vastus medialis (VM) and vastus lateralis (VL) share a significant proportion of synaptic input (Mellor and Hodges, 2005; Laine et al., 2015; Rossato et al., 2024). Consequently, VM and VL are considered a synergistic pair for knee extension and patellar stability (Powers, 2000; Laine et al., 2015). Such shared input limits the degrees of freedom and available motor-control strategies (Milner-Brown et al., 1973; Desmedt and Godaux, 1977, 1978; Marshall et al., 2022; Rossato et al., 2024).

In contrast, recent studies using high-density surface electromyography (HDsEMG) combined with MU decomposition during isometric contractions have revealed that MU discharge behavior in VM and VL is organized into low-dimensional neural structures rather than being governed by a single common drive (Del Vecchio et al., 2023). By applying analytical techniques such as factor analysis (FA) and principal component analysis, approaches previously used to extract muscle synergies from global electromyography (EMG) signals (d’Avella et al., 2003; Tresch et al., 2006), distinct neural modes of MU discharge have been identified within and across these muscles (Del Vecchio et al., 2023). These modes reflect structured common synaptic inputs, where subsets of MUs covary more strongly in discharge rate. Such modes may be differentially weighted to support flexible or muscle-specific strategies (d’Avella et al., 2003; Del Vecchio et al., 2023; Weinman et al., 2024). It is unknown whether humans can voluntarily access or modulate these shared inputs. Direct tests of voluntary dissociation have produced conflicting results. Rossato et al. (2024) showed that even when participants received real-time feedback of MU discharge rates and were explicitly instructed to dissociate VM and VL, they were unable to separate discharge rates of concurrently active MUs across the two muscles. These findings suggest that the presence of multiple neural modes does not necessarily imply voluntary control over them, possibly due to a dominant low-dimensional common drive and constraints of HDsEMG sampling, which is biased toward superficial MUs (Rossato et al., 2024). This raises a key unresolved question: Can humans achieve independent control of MU modes across VM and VL or even within subregions?

To address this question, we combined multiple intramuscular EMG recordings with real-time feedback of root-mean-square (RMS) amplitude and offline MU decomposition while participants explored voluntary strategies to activate different regions of VM and VL. We tested whether humans can (1) dissociate activation of VM and VL using feedback from targeted intramuscular regions, (2) selectively recruit distinct subsets of MUs within proximal versus distal VM, and (3) reproduce this control across repeated trials. We hypothesized that participants would be able to modulate neural inputs across tasks by recruiting distinct sets of MUs but that this capacity would differ across regions.

Our results demonstrate that most participants were able to dissociate activation of the proximal portion of the VM from the remaining synergistic muscle regions, including the distal VM and the VL. In a subset of participants, full dissociation between VM and VL was also achieved. These findings suggest that region-specific modulation of neural input is possible within synergistic muscles but that this capability is constrained by anatomical and neuromechanical factors. This appears to be the first study to combine intramuscular EMG with real-time feedback to show that humans can selectively recruit distinct sets of MUs within the quadriceps. These results reveal a previously underappreciated capacity for selective neuromuscular control, especially in proximal VM.

## Materials and Methods

### Study design

**Figure 1.**
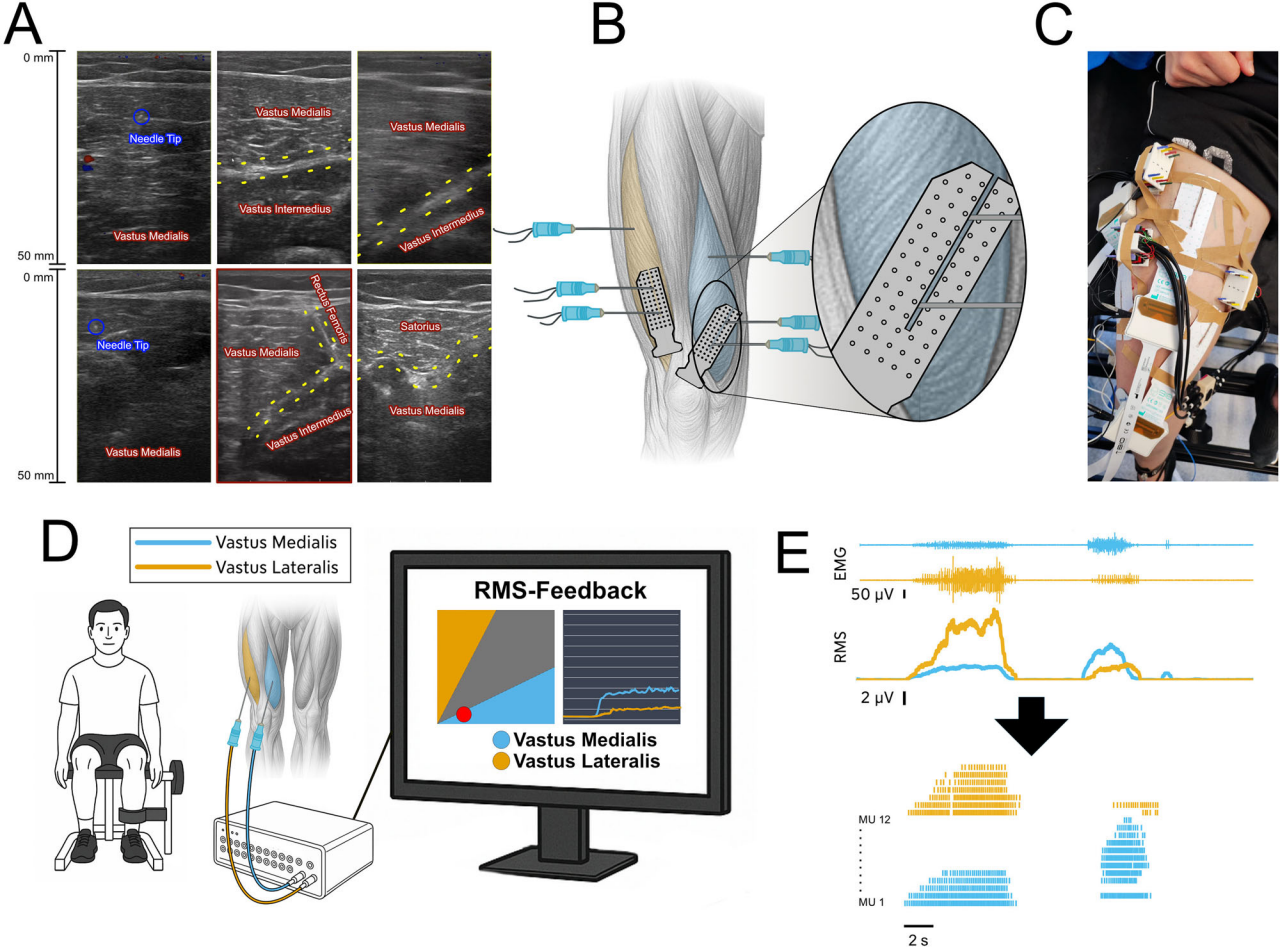
***A***, Anatomical visualization using ultrasound imaging. Six ultrasound images illustrate the anatomical identification and electrode placement within the quadriceps. The two images on the left display the needle tip within the VM during insertion, confirming intramuscular electrode position. The four images on the right depict transverse views of the quadriceps muscles at different depths and locations, highlighting clear separation between the VM, VL, and rectus femoris muscles. Muscle borders are outlined, and muscle labels are overlaid to validate targeted electrode placement. ***B***, Electrode placement for intramuscular and surface EMG recordings. The schematic illustrates the placement of recording electrodes in the right quadriceps. The VL region is highlighted in yellow, and the VM in blue. Three intramuscular electrodes were inserted into each muscle (shown as needle symbols), with two electrodes positioned distally and one proximally in VM. HDsEMG grids were placed over the distal VM and VL regions. In VM, a small gap was cut into the grid to allow placement of the underlying intramuscular electrodes. This configuration ensured simultaneous recording of surface and deeper intramuscular activity. The proximal intramuscular electrode in VM was always positioned outside the grid area to capture regional differences in muscle activation. ***C***, Exemplary final electrode setup on a leg. This photograph shows the complete electrode configuration on a participant’s leg. All intramuscular and surface EMG electrodes are visible, including grids over the VM and VL, as well as one placed on the rectus femoris. Although a grid electrode was placed over rectus femoris, data from this muscle were not analyzed in the present study. The image provides a practical overview of the real-world implementation of the setup and demonstrates the location of the electrodes across quadriceps muscles. ***D***, Schematic representation of the experimental setup. This schematic illustrates the experimental setup during data collection. Each participant was seated on an isokinetic dynamometer with the instrumented leg attached to the lever arm to ensure isolated isometric conditions. The screen was placed in front of the participant to display real-time EMG feedback. Two visualizations are shown: a 2D scatterplot of RMS amplitudes for VM versus VL and a time-series plot of RMS activity. ***E***, Example of resulting processed signal. This panel illustrates EMG recordings and the associated processing steps for one channel from each of VM (blue) and VL (yellow). The top trace shows the interference intramuscular EMG signals during alternating VM- and VL-focused tasks. Below, the RMS amplitude envelopes for each muscle are shown. The bottom trace displays the spike trains of identified MUs obtained through offline decomposition. Each row represents a single MU, with vertical ticks marking discharge times.

This study evaluated the ability of participants to modulate the neural input to the VM and VL, building on previous findings from HDsEMG and FA of MU discharge rates (Del Vecchio et al., 2023; Levine et al., 2023). Each participant completed a single 30 min experimental session. Rather than following a prescribed protocol, participants were encouraged to explore voluntary strategies to achieve selective activation of either the VM or VL while maintaining low activation levels to facilitate accurate MU decomposition. All tasks were performed in a seated position on an isokinetic dynamometer, with the instrumented leg attached to the lever arm (Fig. 1*D*).

A VM-focused task was defined as an attempt to increase the EMG feedback signal from VM while keeping VL activation low. Conversely, a VL-focused task was defined as increasing the EMG feedback signal from VL while minimizing VM activation. Participants were informed that the visual feedback indicated the relative activity levels of VM and VL and were instructed to modulate the amount of activity so that in the target muscle increased relative to the nontarget muscle. To avoid confounding influences from whole-body bracing or remote muscle coactivation, experimenters monitored global posture and visually inspected noninstrumented muscles. No overt coactivation outside the quadriceps was observed in any of the included trials.

Throughout the session, participants received online feedback based on the RMS amplitude of intramuscular EMG signals, displayed either as time-series plots or as two-dimensional plots of VM and VL activity. Participants were told that these signals corresponded to the activity level of each individual muscle and were instructed to maintain low activation levels. Our primary outcomes of the study were (1) selective activation of VM or VL during real-time tasks and (2) the identification of MU activity during VM- and VL-focused tasks, assessed via offline decomposition of intramuscular and surface EMG signals. This exploratory structure was chosen to allow participants to discover strategies that increased the feedback signal, in line with operant-conditioning approaches used to modulate spinal and cortical activity.

### Participants

Ten healthy individuals (mean age 29 ± 5.4 years; six males, four females) took part in the study. All were free from neurological or musculoskeletal disorders affecting the lower limbs. Written informed consent was obtained from all participants prior to testing. The study protocol was approved by the ethics committee of the Friedrich-Alexander University Erlangen–Nürnberg (ethics vote 25-37_2-S) and was conducted in accordance with the Declaration of Helsinki.

### Experimental protocol

Fine-wire intramuscular recordings were obtained using bipolar hook-wire electrodes (Spes Medica). Each electrode comprised a stainless-steel sensor wire (0.11 mm diameter) insulated with PTFE and preshaped into a small hook at the tip. Electrodes were inserted using a sterile 25 G (0.50 mm) stainless-steel hypodermic needle, through which the wire was advanced and deployed as the needle was withdrawn, allowing the hooked end to anchor securely within the muscle.

Electrodes were inserted into both VM and VL using standard anatomical landmarks. For VM, the insertion site was identified by locating the medial border of the patella and tracing the VM muscle proximally along the medial thigh. For VL, insertions were guided by the lateral border of the patella and the line between the greater trochanter and the lateral femoral condyle, following the lateral contour of the thigh. After approximate placement was established, the needle was advanced into the muscle belly along the chosen trajectory.

All insertions were guided and verified using real-time ultrasound imaging (Fig. 1*A*). Although it was not possible to visualize the wire itself, the needle tip and muscle borders were clearly identifiable, allowing accurate placement within the intended muscle and avoiding penetration of adjacent muscles. Gentle needle manipulation further confirmed correct positioning prior to deployment of the wire. This procedure minimized cross talk from neighboring muscles and ensured that the intramuscular recordings reflected activity from the targeted region. In each muscle, three electrodes were inserted to capture regional differences in activation (Fig. 1*B*): two were positioned distally and one proximally.

**Figure 2.**
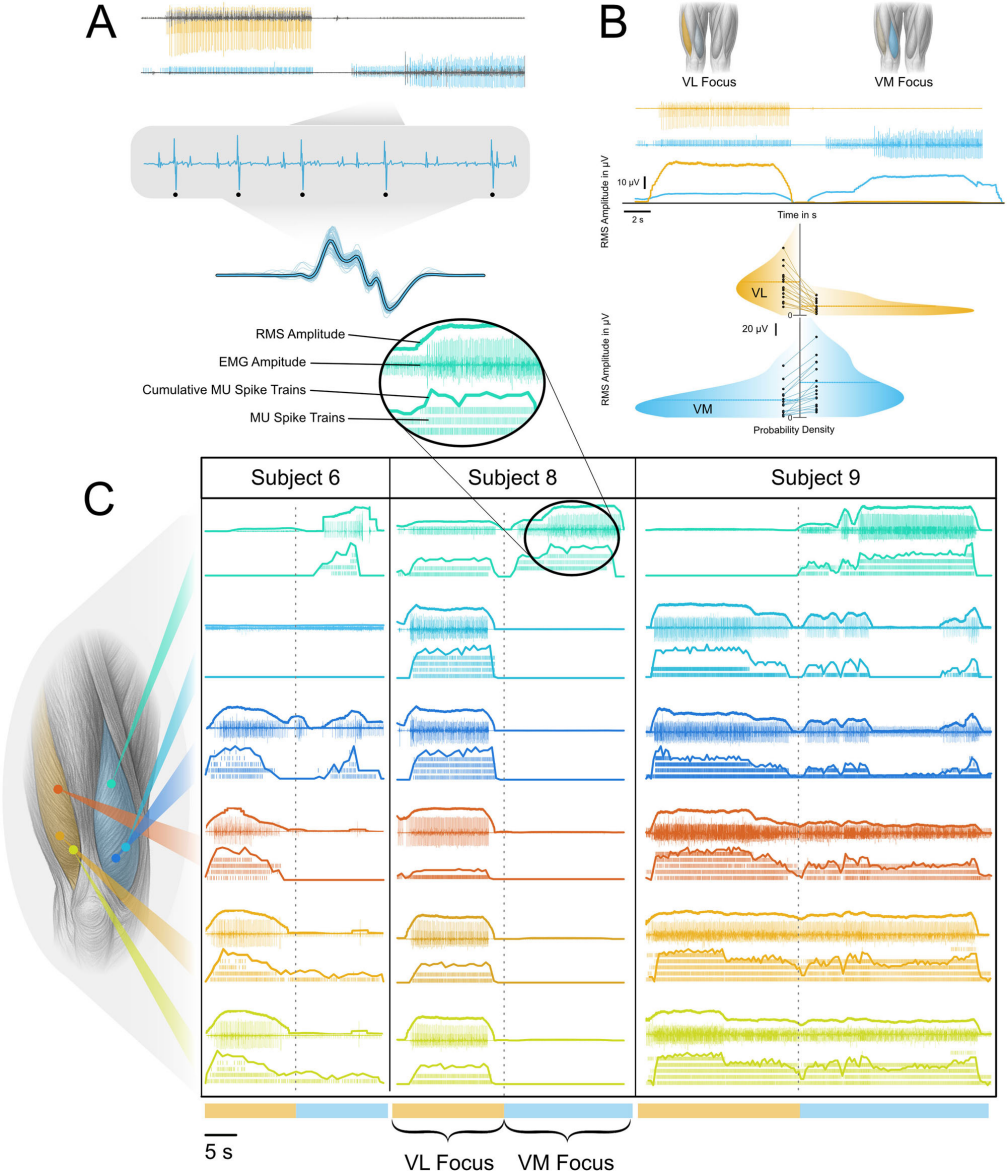
***A***, Validation and visualization of intramuscular EMG decomposition. The top part of the figure shows intramuscular EMG signals from VM (blue) and VL (yellow) channels, overlaid with the residual EMG after decomposition (gray), highlighting the portion of the signal not explained by the identified MU spike trains. A zoomed section of the EMG trace displays individual MUAPs, with five discharges of the same MU highlighted to visualize their consistent waveform. The bottom part of the figure shows the STA for the selected MU with the identified waveforms superimposed, and the resulting average MUAP shape is indicated by the thick line. ***B***, Task-specific modulation of EMG and RMS signals in VM and VL. The top row illustrates the instructed task. VM and VL are alternately highlighted to indicate periods where participants were instructed to focus on selectively activating either muscle. Below, intramuscular EMG signals from VM (blue) and VL (yellow) are shown over time, followed by the corresponding RMS amplitude traces. The bottom panels summarize group-level results from all participants (*N* = 9), displaying the probability density distributions of RMS amplitudes for VM and VL separately in the corresponding colors. The distributions are shown for VM-focused and VL-focused tasks for each muscle. ***C***, Overview of multichannel and multisubject MU recruitment. EMG activity across all intramuscular channels for three representative participants. The colored traces are matched to the electrode locations. The EMG traces (overlaid with the RMS envelopes) are shown for each participant during both task conditions. Below the raster plots visualize the MU spike trains, with each row representing a unique unit. Cumulative discharge rates are overlaid on top to reflect overall modulation of recruitment across tasks.

To complement intramuscular recordings, we placed HDsEMG grids over the skin above the distal intramuscular electrode in both VM and VL. The intramuscular wires were inserted through small cutouts in the grids (Fig. 2*B*,*C*).

Participants remained seated throughout the experiment in an isometric dynamometer instrumented with a three-dimensional load cell. The tested leg was attached to the lever arm to prevent unintended movement, and a small foot support platform was added to stabilize the ankle. This platform was not mechanically coupled to the force transducer, which resulted in partial underestimation of vertical force components.

The experiment began with a brief exploration phase (∼5 min) during which participants were allowed to make small voluntary adjustments to hip rotation, knee angle, or baseline muscle pretension to discover a strategy for modulating VM and VL independently. Once formal trials began, the posture was required to remain fixed, and all trials were visually monitored to ensure that joint angle, leg orientation, and electrode geometry remained constant across conditions.

During the experiment, participants received continuous visual feedback of the RMS amplitude of the VM and VL intramuscular EMG signals. They were instructed to increase the amplitude of the feedback signal of either VM or VL while minimizing activation of the nontarget muscle, depending on the task segment (VM-focused vs VL-focused task). This structure enabled participants to explore individualized activation strategies without imposing external force targets. They were also instructed to include a brief relaxation period between task segments to ensure full derecruitment of MUs.

Across the session (∼30 min), participants performed multiple VM- and VL-focused attempts. A trial was defined as one continuous recording containing both a VM-focused and a VL-focused segment. The experimenter initiated formal recordings when participants demonstrated a clear and repeatable ability to alternate between VM and VL activation. At least two such trials were collected from each participant, as required for the repeatability and MU tracking analyses. A trial was considered successful when participants showed consistent and voluntary modulation of muscle activity, as reflected in the real-time EMG feedback.

### Force-direction recording and analysis

To complement the EMG recordings, three-dimensional forces applied to the dynamometer were recorded during the selective activation tasks. The instrumented leg was fixed to the lever arm of the isokinetic dynamometer, constraining movement in the lateral, vertical, and anterior–posterior directions. To stabilize the foot and facilitate selective activation, a small support platform was added beneath the foot. Because this platform was not mechanically coupled to the dynamometer’s force transducer, the absolute magnitude of the vertical force was underestimated. Nevertheless, vertical force contributions were consistently captured and were clearly visible in the recordings.

Force signals were recorded continuously during VM-focused and VL-focused task segments. For each participant and condition, forces were averaged across repetitions to obtain a single representative force vector per task. The coordinate system was defined such that the *x*-axis represented lateral force, the *y*-axis represented vertical force, and the *z*-axis represented forward–backward force.

To focus on differences in force orientation independent of magnitude, the force vectors were normalized to unit length prior to directional analyses. In this normalization, each force vector was divided by its Euclidean norm so that all vectors had a magnitude of one. Angular separation between VM- and VL-focused force directions was computed within each participant using the angle between the corresponding unit vectors. To characterize task-dependent changes in force orientation, difference vectors were computed as the vector subtraction between VL-focused and VM-focused unit vectors for each participant.

### Control ramp contractions

To determine whether the regional dissociation observed during the EMG feedback tasks also emerged under conventional loading conditions, participants performed additional isometric ramp contractions without visual EMG feedback. These ramps served as a control condition intended to capture the natural concurrent modulation of VM and VL during standardized tasks.

Participants were positioned identically to the main experiment, with all EMG electrodes in place and the knee fixed at 45° flexion. They were instructed to follow a visual force trajectory and perform two linear ramps, reaching 15 and 30% of maximal voluntary contraction (MVC) force, each followed by a 30 s plateau. Every ramp condition was repeated three times. No instructions regarding selective muscle activation were provided.

EMG signals were recorded from all intramuscular and HDsEMG channels. For each ramp trial, RMS amplitudes were extracted from proximal and distal VM and VL channels, and pairwise Pearson’s correlation coefficients were computed over the ramp-up phase. These correlations provided an estimate of the default coupling of EMG activity across VM and VL regions during typical isometric contractions, allowing comparison with the selective activation tasks.

### EMG feedback

EMG feedback was provided using a custom Python interface that displayed either (1) a two-dimensional plot of RMS amplitude for VM versus VL or (2) a scrolling time-series trace of both signals (Fig. 1*D*). RMS amplitudes were computed continuously using a 300 ms sliding window, updated at 20 Hz (i.e., every 50 ms), resulting in overlapping windows with 250 ms overlap between successive estimates. The software pipeline introduced <20 ms of computational delay, whereas the RMS window imposed an inherent causal smoothing delay corresponding to the preceding 300 ms of activity. Thus, the feedback provided a brief history of the ongoing contraction. This latency was stable across the session and was sufficiently brief to allow participants to adjust activation strategies in near real time.

To facilitate interpretation, each channel was scaled dynamically so that the feedback emphasized the relative difference in activity between VM and VL rather than absolute RMS magnitude. This normalization allowed participants to observe increases in the target muscle while keeping the nontarget muscle at low activation levels, independent of absolute differences in signal amplitude across channels or subjects.

All EMG signals (intramuscular and HDsEMG) were recorded using the OT Bioelettronica Quattrocento system, sampled at 10,240 Hz and hardware-filtered between 10 and 5,000 Hz prior to RMS computation.

### Data processing

RMS amplitudes were computed from all intramuscular EMG channels for every trial. For each participant, trial, and intramuscular channel, we calculated the mean RMS amplitude of VM and VL during the VM-focused and VL-focused task segments (*R*_VM, VMf_, *R*_VL, VMf_, *R*_VM, VLf_, *R*_VL, VLf_). To quantify how strongly a given VM–VL channel pair expressed selective activation, we defined a separation index as follows:

$$S=(R_{\mathrm{VM},\mathrm{VMf}}-R_{\mathrm{VL},\mathrm{VMf}})+(R_{\mathrm{VL},\mathrm{VLf}}-R_{\mathrm{VM},\;\mathrm{VLf}}).$$

Higher values of *S* indicate greater activation of the target muscle and stronger suppression of the nontarget muscle across the two task segments.

For each trial, this index was computed for all possible pairs of one VM intramuscular channel and one VL intramuscular channel. The VM–VL channel pair with the highest separation index within a trial was selected for analysis. The corresponding *S* value defined the trial’s separation score. Trials were ranked for each participant according to their separation score, and the two trials with the highest S values were selected for further analysis, provided that each task segment contained at least 3 s of stable EMG activity. These two trials formed the basis for assessing repeatability of task-dependent activity. MU decomposition was first attempted on these trials. If one of the selected trials did not meet decomposition quality criteria (see below), additional trials were evaluated in a descending order of separation score using the same procedure until two usable trials were identified. Participants for whom no two qualifying trials could be obtained were excluded from MU analyses.

Selected data segments were subjected to offline MU decomposition using EMGLab, a validated manual decomposition toolbox (McGill et al., 2005), running in MATLAB (Version R2024b, The MathWorks). MU spike trains were manually reviewed and edited to ensure accurate identification of discharge times and assigned to the respective muscle. Spike rasters were inspected to determine whether individual MUs were active during VM- or VL-focused tasks. Discharge rates and cumulative spike trains were computed and compared between VM- and VL-focused strategies (Fig. 1*E*).

To assess decomposition quality, reconstructed EMG signals were generated from identified spike trains and compared with the original recordings (Fig. 2*A*). The residual EMG, defined as the difference between original and reconstructed signals, was expressed as a percentage of total EMG power. Only data segments with residual EMG power <30% were retained for further analysis (de Luca et al., 2015; Negro et al., 2016a), indicating that the extracted MUs accounted for most of the signal variance.

MUs identified in the selected trials were next examined for cross-trial correspondence. For each MU, a spike-triggered average (STA) of the intramuscular EMG was computed for both trials, and units were first matched using the temporal similarity of these waveforms. Intramuscular STA correlations exceeding *r* > 0.90 were taken as evidence that the same MU was present in both trials. When the intramuscular MU action potential (MUAP) waveform changed substantially across trials, typically reflecting small shifts in electrode-fiber geometry, STA matching alone was no longer reliable. In these cases, MU identity was validated using HDsEMG-derived spatial MUAP maps generated by projecting the intramuscularly identified spike times onto the concurrently recorded HDsEMG grid. Units were therefore accepted as matches when the spatial MUAP maps showed a cross-trial correlation greater than *r* > 0.85. To ensure conservative matching, the trial with the smaller MU yield was used as the reference within each participant and muscle.

For the compartmentalization analysis within each trial, pairwise Pearson’s correlation coefficients were computed between proximal and distal VM channels and between VM and VL channels. Because correlation coefficients are not normally distributed, all values were Fisher *z*-transformed prior to statistical testing, whereas absolute *r* values are reported descriptively.

### Statistical analysis

All statistical analyses were performed in MATLAB (R2024b). Because each participant contributed repeated observations across trials, all linear mixed-effects models (LMEs) included a random intercept for Subject.

Differences in RMS amplitude were evaluated using an LME with Muscle (VM, VL) and Task (VM-focused, VL-focused) as fixed factors. The Muscle × Task interaction tested whether each muscle increased activation preferentially during its respective task. Residuals were examined using the Lilliefors test; although mild deviations from normality were present, no transformation was applied because LMEs are robust to such violations. Descriptive values are reported as mean ± SD.

MU counts were analyzed using the same model structure, testing whether the number of active units differed systematically between muscles and tasks. Although counts are reported as integers, inspection of residuals indicated that a LME provided an adequate fit without requiring Poisson or binomial link functions.

Task-specific MU recruitment was quantified using a uniqueness proportion for each participant and muscle, defined as the fraction of units active exclusively during the target-focused task. Uniqueness values were averaged across the two decomposed trials per participant and compared between VM and VL with a paired *t* test. Occurrences of “cross-task unique” units, present only in the nontarget task, were summarized descriptively.

For the compartmentalization analysis, pairwise Pearson’s correlation coefficients were computed for selected intramuscular channel pairs within each trial during both EMG feedback and ramp-contraction conditions. Because correlations are not normally distributed, all statistical comparisons were performed on Fisher *z*-transformed values. Within-condition differences (e.g., proximal VM vs distal VM) and between-condition differences (feedback vs ramp) were tested using paired *t* tests applied to subject-level averages. Absolute correlation coefficients are reported for interpretability; inferential statistics used the transformed data.

For force-direction analyses, angular separations between normalized force vectors were computed within each participant. One-sample *t* tests and complementary Wilcoxon signed-rank tests were used to assess whether VM–VL angular differences differed from zero. Paired *t* tests were used to compare angular deviations of VM- and VL-focused force vectors relative to control conditions. Consistency of task-dependent shift in force direction across participants was assessed using a multivariate Hotelling’s *T*^2^ test, which tests whether the mean three-dimensional shift vector differs from the null vector (0,0,0) across participants, thereby assessing group-level consistency in force-direction changes. This analysis was supplemented by component-wise one–sample *t* tests on the *x*-, *y*-, and *z*-components.

For LMEs, effect sizes are reported as partial eta squared (*η*^2^*p*), calculated from the *F* statistic and associated degrees of freedom. For paired and one-sample *t* tests, Cohen’s *d* (dz for paired comparisons) was computed as *t*/√*n*, where *n* denotes the number of subjects. For correlation analyses, effect sizes for paired comparisons of Fisher *z*-transformed values are reported as Cohen’s dz.

Statistical significance was defined as *p* < 0.05.

## Results

### EMG amplitude analysis

As an initial assessment of the ability to activate the VM and VL selectively, we analyzed the EMG amplitude by computing normalized RMS amplitudes of the intramuscular EMG recordings across the different tasks. Subsequently (offline), we extracted two trial segments from every participant that included the sequential control of VM- and VL-focused tasks. For each participant, one VM channel and one VL channel were selected based on the largest task-dependent RMS difference (see Materials and Methods). The channel showing the clearest modulation in VM was typically the proximal electrode (7/9 participants), whereas all VL channels exhibited similar activity. The remaining two participants showed clear task-dependent modulation in both proximal and distal VM channels.

Participants consistently demonstrated task-dependent modulation of VM and VL activity (Fig. 2*B*). Across all participants and trials, RMS amplitude was highest in VM during VM-focused tasks (56 ± 39 µV) and highest in VL during VL-focused tasks (57 ± 34 µV), with markedly lower amplitudes during the nontarget tasks (VM during VL-focus, 25 ± 26 µV; VL during VM-focus, 15 ± 12 µV). An LME confirmed significant main effects of Muscle (*F*_(1,68)_ = 10.92; *p* = 0.0015; *η*^2^*p* = 0.14) and Task (*F*_(1,68)_ = 19.29; *p* < 0.0001; *η*^2^*p* = 0.22), as well as a strong Muscle × Task interaction (*F*_(1,68)_ = 29.43; *p* = 8.3 × 10^−^⁷; *η*^2^*p* = 0.30). These statistics confirm that participants increased VM activity specifically during VM-focused tasks and increased VL activity during VL-focused tasks.

Model residuals showed mild deviations from normality (Lilliefors *p* = 0.012), which is expected for EMG amplitude data, but no heteroscedasticity or influential outliers were detected. Together, these results demonstrate robust, task-dependent modulation of VM and VL across participants, providing a reliable basis for subsequent MU-level analyses.

### MU recruitment

The intramuscular EMG signals from the same trials used in the RMS analysis were decomposed into MU spike trains. For each participant, decomposition was first attempted on the two trials that showed the clearest VM–VL alternation in the RMS signals. When decomposition quality in one of these trials did not meet the residual-power criterion (e.g., due to unstable baseline, higher activation levels, or movement-related artifacts), we examined additional trials from the same participant. This ensured that trial selection for decomposition was based on both the presence of strong task-dependent modulation and the signal quality required for reliable MU identification.

This procedure identified two usable decomposed trials from eight participants. For one participant, no available trial segments met both criteria simultaneously; that is, although RMS separation was evident, none of the trials yielded intramuscular EMG of sufficient quality to pass the residual-power threshold. Decomposition quality was quantified by the residual EMG power after subtracting the reconstructed MU activity from the original signal, and only segments with residual power below 30% were retained (de Luca et al., 2015; Negro et al., 2016a). Residual power averaged 18.0 ± 5.8% for the included recordings.

**Figure 3.**
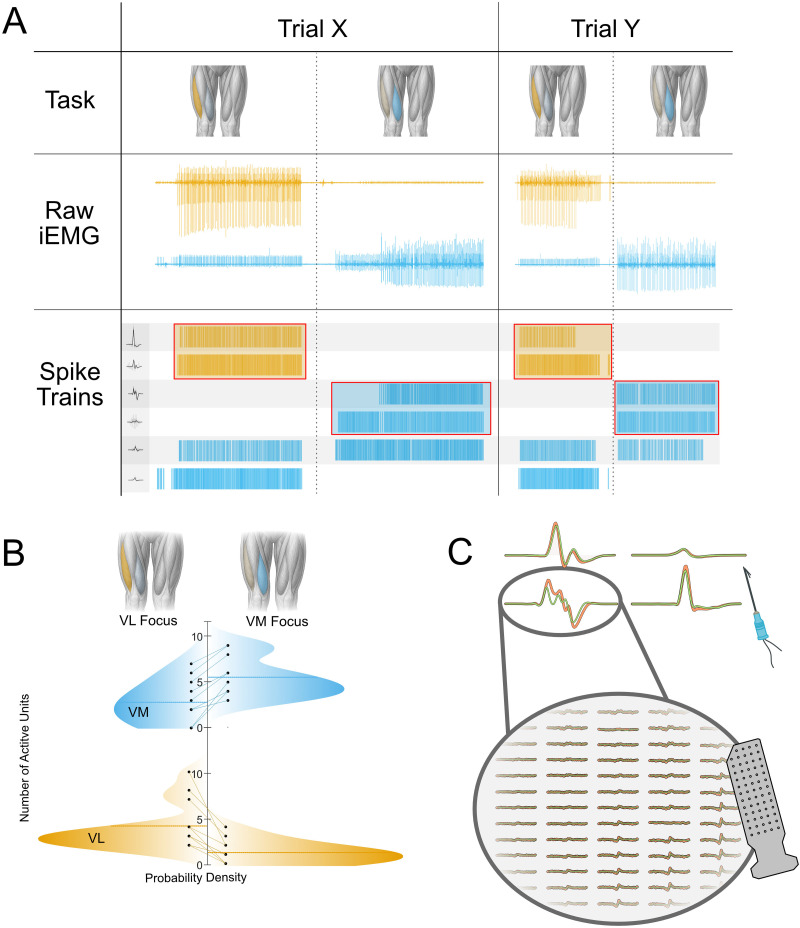
***A***, Comparison of neural drive and MU activity across two trials in one participant. The top row illustrates the instructed task sequence for each trial, highlighting periods of VL-focused (orange shading) and VM-focused (blue shading) activity. Below, intramuscular EMG signals are shown for one channel each from VL and VM, plotted over time and aligned with the task segments. The bottom row displays the corresponding spike trains of all identified MUs, color-coded by muscle (blue for VM, orange for VL), revealing the change in MU recruitment between VM- and VL-focused segments and the consistency across trials. ***B***, Distribution of active MUs across tasks. Probability density plots summarize the number of active MUs identified across all analyzed trials. For each participant, two trials per task were selected based on clear task-dependent modulation observed in RMS signals. Separate distributions are shown for VM-focused and VL-focused tasks, with data color-coded by muscle (blue for VM, yellow for VL). The *y*-axis represents the number of active MUs per trial, while the *x*-axis indicates the estimated probability density. Horizontal lines mark the mean number of active units for each distribution. ***C***, MUAP tracking using intramuscular and surface electrode recordings. Examples of MUAPs from four representative MUs are shown. For each unit, STAs of the intramuscular EMG obtained from two different trials are overlaid (green and orange traces), illustrating the reproducibility of MUAP waveform shape across repetitions. In cases where intramuscular MUAP shapes differed slightly between trials, MU identity was confirmed using spatial MUAP maps derived from the concurrently recorded HDsEMG. These maps were computed by projecting intramuscularly identified discharge times onto the HDsEMG grid, to demonstrate reliable cross-trial MU matching.

Figure 3*A* illustrates a representative example from two trials of one participant. The top row indicates the instructed task segment (VM- or VL-focused task), followed by the EMG traces from VM and VL. The extracted discharge times of decomposed MUs are shown in the bottom panel. A distinct subset of MUs was recruited in VM (marked red) during the VM-focused task with minimal VL activity. This pattern was reversed during the VL-focused task. This pattern was stable across trials, with the same MUs consistently activated during VM- or VL-focused tasks. All channels from three representative participants are displayed in Figure 2*C*.

Across all decomposed trials, MU activity mirrored the task-dependent modulation observed in the RMS amplitudes. VM recruited more MUs during VM-focused tasks (6.2 ± 2.4 units) than during VL-focused tasks (2.9 ± 2.2 units), whereas VL showed the opposite pattern, with greater recruitment during VL-focused tasks (4.1 ± 2.3 units) than during VM-focused tasks (1.6 ± 1.4 units). An LME with fixed factors Muscle and Task revealed a significant main effect of Task (*F*_(1,60)_ = 12.08; *p* = 0.00095; *η*^2^*p* = 0.17) and, critically, a strong Muscle × Task interaction (*F*_(1,60)_ = 31.96; *p* = 4.6 × 10^−^⁷; *η*^2^*p* = 0.35), indicating that each muscle recruited more MUs when it was the focus of the selective contraction. The main effect of Muscle was not significant (*F*_(1,60)_ = 2.73; *p* = 0.104). Residuals showed moderate deviations from normality (Lilliefors *p* = 0.0012), expected for MU count data, but variance was homogeneous across conditions, and linear mixed models are robust to such deviations. Although most participants modulated MU activity in a task-dependent manner, complete derecruitment of the nontarget muscle was rare and observed only in isolated cases. These differences are visualized in Figure 3*B*.

To quantify the selective activation of MUs during each task, we computed the proportion of MUs that were active only during the target-focused task within each muscle (i.e., VM units active in VM-focused but not VL-focused, and VL units active in VL-focused but not VM-focused). Uniqueness was computed at the participant level by averaging across the two decomposed trials.

Across the eight participants, the VM showed a mean task-specific uniqueness of 0.62 ± 0.22, whereas the VL showed a similar uniqueness of 0.65 ± 0.21. A paired comparison revealed no significant difference between VM and VL uniqueness (*t*_(7)_ = −0.30; *p* = 0.774; *d* = 0.11), indicating that both muscles exhibited a comparable degree of task-specific recruitment. Although participants were able to recruit partially distinct subsets of MUs for VM- and VL-focused tasks, complete segregation of MU activity was not observed.

We also examined whether MUs were active only during the task focused on the other muscle. This “cross-task unique” activity was observed in a subset of participants. MUs in VM that were active exclusively during VL-focused tasks occurred in four of eight participants, whereas VL uniquely active MUs during VM-focused tasks were rare and occurred in only one of eight participants. These findings show that selective modulation was not restricted to enhancing activity in the target muscle; in some cases, participants recruited distinct MUs in the nontarget muscle when adopting specific strategies.

Because these interpretations rely on the stability of MU identification across trials, we next quantified the reproducibility of task-dependent recruitment patterns. MUs were tracked across the two decomposed trials obtained from each of the eight participants (Fig. 3*C*). Units were first matched using a temporal criterion based on the cross-trial correlation of the intramuscular STAs; correlations exceeding *r* > 0.90 were taken to indicate the same MU. In trials where the intramuscular MUAP waveform changed substantially, STA correlations no longer provided a reliable match. In those cases, MU identification relied on HDsEMG-derived spatial MUAP maps, which are more stable across shifts in posture and electrode location, and units were considered matches when the spatial correlation exceeded *r* > 0.85. To avoid inflating mismatch rates due to differences in decomposition yield, the trial containing the smaller number of MUs was used as the reference within each participant and muscle.

Across all participants, 45 MUs in VM and 27 MUs in VL were available for cross-trial comparison. Of these, 44 VM units (98%) and 24 VL units (88%) met the matching criteria and were consistently identified across both trials. Tracked units also showed stable activity: units that were unique to VM- or VL-focused tasks in one trial displayed the same task-dependent classification in the second trial, whereas shared units remained active across tasks in both trials. These findings demonstrate that the strategies used to differentiate VM- and VL-focused tasks were highly reproducible across repeated attempts.

### VM compartment analysis

**Figure 4.**
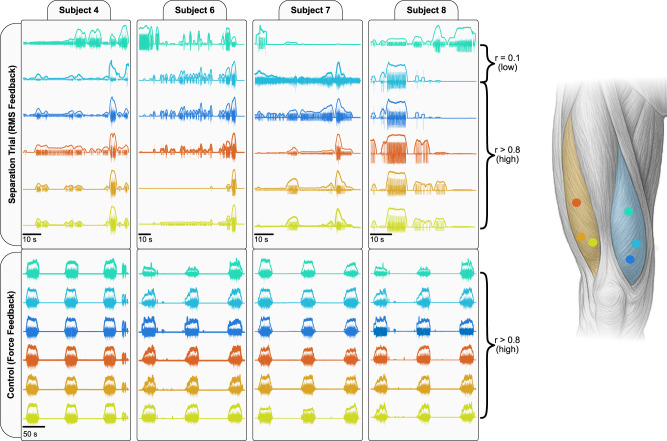
Overview of intramuscular EMG activity in VM and VL across four subjects. This panel shows representative intramuscular EMG recordings from both the feedback condition (***A***, RMS-guided selective activation) and the control condition (***B***, force feedback) across four participants. EMG signals from all implanted channels (color-coded) are displayed for each participant. EMG traces are overlaid with their respective RMS amplitude and highlight differences in concurrent modulation across channels. All channels were tightly coupled in the control condition, whereas proximal VM channels often exhibit independent modulation relative to distal VM and VL in the feedback condition. On the right, an anatomical schematic of the anterior thigh illustrates the locations of all intramuscular electrodes. Colored dots correspond to specific EMG channels plotted on the left, enabling anatomical interpretation of the spatial activation differences observed across conditions.

Inspection of intramuscular EMG recordings revealed consistent regional differences in activity across participants. Representative examples from a selective activation trial and a control ramp contraction are shown in Figure 4. During EMG feedback trials, distal VM and VL channels typically exhibited concurrent amplitude modulation, whereas the proximal VM channel often displayed a distinct temporal profile, suggesting reduced coupling with the distal regions. In contrast, during standard ramp contractions without EMG feedback, activity across all channels appeared highly synchronous.

**Figure 5.**
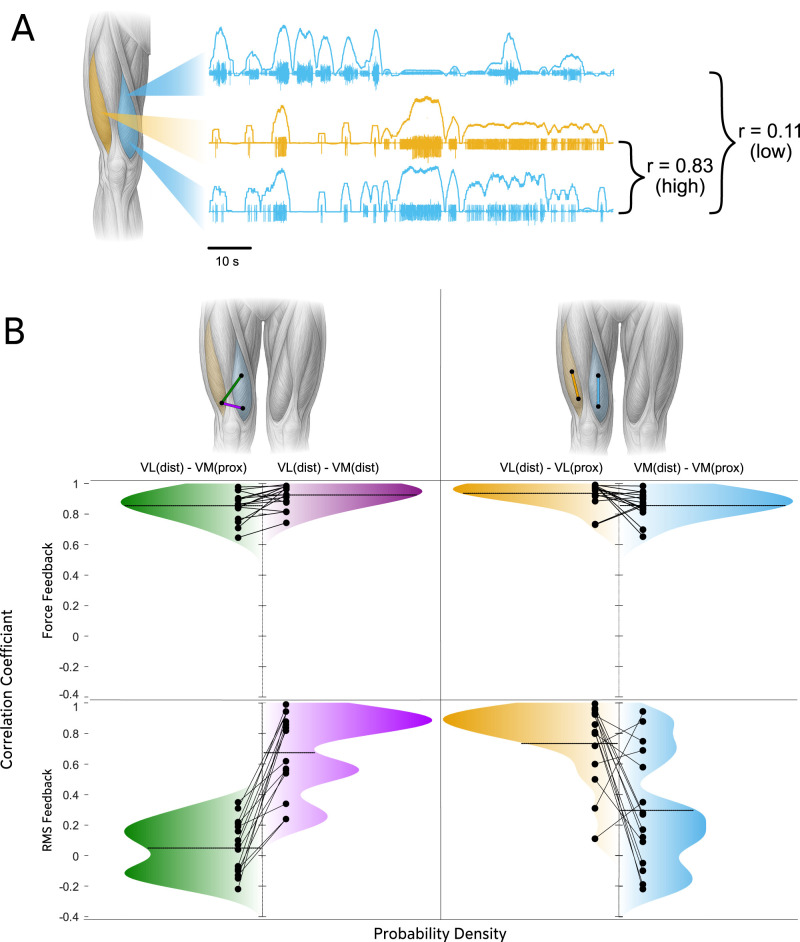
***A***, Visualization of regional EMG recording and correlation analysis. A schematic of the thigh shows the locations of VL (yellow) and VM (blue). Intramuscular EMG recordings acquired concurrently from different muscles and regions (distal and proximal) of the muscle are displayed next to the muscle. Above each EMG trace, the RMS signals are displayed to illustrate the amplitude modulation over time. Correlation coefficients demonstrate a high correlation (*r* = 0.83) between VL and distal VM signals but a lower correlation (*r* = 0.11) between distal and proximal regions of VM. ***B***, Distributions of RMS correlation coefficients across muscle regions. This figure illustrates the distributions of correlation coefficients computed from RMS signals recorded across different quadriceps regions during both the EMG feedback condition and the ramp contractions (force feedback). Each subplot displays a continuous probability density plot, where the vertical axis represents correlation values and the horizontal axis indicates probability density. The top row presents the distributions from the ramp contractions (15 and 30% MVC ramp contractions with force feedback), whereas the bottom row displays the corresponding distributions from the EMG feedback task. Each column shows a specific pairwise comparison: (1) distal VL versus proximal VM, (2) distal VM versus distal VL, (3) distal versus proximal VL, and (4) distal versus proximal VM.

These qualitative observations motivated a quantitative assessment of functional coupling between muscle regions. Because correlation coefficients capture the similarity of temporal modulation across channels, they provide a measure of how strongly different compartments are comodulated over time. During the EMG feedback condition (Fig. 5), clear regional differences emerged. Distal VM and distal VL showed strong comodulation (mean *r* = 0.69 ± 0.19), whereas proximal VM exhibited substantially weaker coupling with both distal VM (0.35 ± 0.26) and distal VL (0.13 ± 0.15). Statistical comparison of *z*-transformed values confirmed that proximal VM was significantly less correlated with distal VM than distal VM was with distal VL (*t*_(7)_ = −2.42; *p* = 0.046; *d* = 0.86) and was markedly less correlated with distal VL (*t*_(7)_ = −4.75; *p* = 0.0021; *d* = 1.68). These results indicate that proximal VM displays partially independent modulation relative to the distal regions of VM and VL during selective activation with EMG feedback.

In contrast, correlations were uniformly high across all channel pairs during standard isometric ramp contractions without EMG feedback (all means *r* > 0.85; Fig. 5). Under these conditions, correlations between proximal and distal VM (0.86 ± 0.08) and between distal VM and distal VL (0.92 ± 0.07) did not differ significantly (*t*_(7)_ = −1.97; *p* = 0.0901; *d* = 0.69). Although proximal VM was still slightly less correlated with distal VL (0.85 ± 0.08) than distal VM was with distal VL, this difference was smaller than in the feedback condition (*t*_(7)_ = −2.55; *p* = 0.0381; *d* = 0.90).

Direct comparison between conditions revealed that correlations were systematically lower during EMG feedback than during ramp contractions across all channel pairs (all *p* < 0.05 after Fisher *z*-transform). The largest condition effect was observed for the proximal VM-distal VL pair (*t*_(7)_ = −8.64; *p* = 0.0001; *d* = 3.06), followed by proximal VM-distal VM (*t*_(7)_ = −7.72; *p* = 0.0001; *d* = 2.73). These results show that feedback-driven selective activation amplifies regional differences in EMG amplitude that are largely absent during conventional force-controlled contractions.

Overall, these analyses show that selective activation induces region-specific modulation within VM not expressed during conventional contractions, supporting a flexible rather than fixed organization of quadriceps motor control.

### Task-dependent modulation of force direction

**Figure 6.**
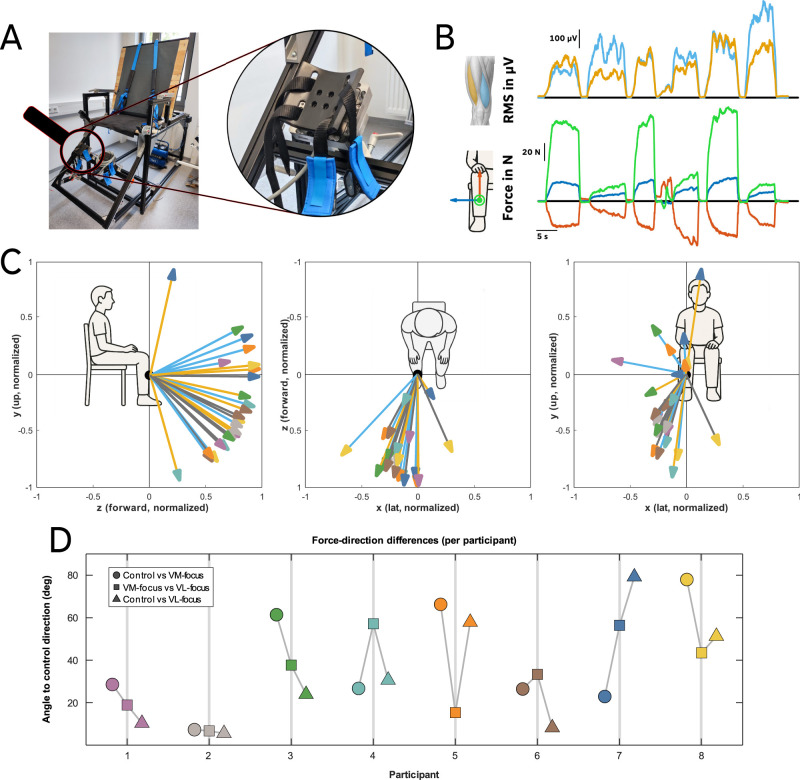
***A***, Photograph of the dynamometer with the integrated three-dimensional force sensor. The lower leg was strapped to the lever arm to restrict movement in the side-to-side (*x*), upward–downward (*y*), and forward–backward (*z*) directions. ***B***, Representative recording from a single participant. The top panel shows the RMS amplitude of intramuscular EMG signals from VM and VL during alternating VM- and VL-focused task segments. The lower panel shows the simultaneously recorded three-dimensional force components (green: anterior–posterior; blue, lateral; red, vertical). ***C***, Two-dimensional projections of force vectors recorded during VM- and VL-focused conditions. Each arrow represents one exemplar force vector per participant (blue, VM-focused; orange, VL-focused; gray, control ramp contraction). Arrow tips are color-coded by participant. The plots show normalized unit vectors projected onto the *y*–*z* (vertical–anterior–posterior), *x*–*z* (lateral–anterior–posterior), and *x*–*y* (lateral-vertical) planes, emphasizing task-dependent differences in force direction. ***D***, Participant-wise angular differences between force directions. The angular deviation of the VM-focused and VL-focused force vectors relative to the control ramp direction is shown for each participant. Circles indicate the angle between control and VM-focused contractions, triangles indicate the angle between control and VL-focused contractions, and squares indicate the angular separation between VM- and VL-focused force vectors. Marker colors correspond to participant identity and match the arrow-tip colors shown in panel ***C***.

The lower leg was attached to the dynamometer to minimize movement in the lateral (*x*), vertical (*y*), and anterior–posterior (*z*) directions (Fig. 6*A*). During the task, several participants reported that selective activation of the VM or VL was facilitated by applying downward pressure through the foot.

To assess whether selective activation of VM and VL was accompanied by systematic changes in force application, we analyzed the direction of the net force applied to the dynamometer during VM- and VL-focused tasks (Fig. 6*C*). For each participant, force vectors were normalized to unit length to isolate directional differences. The angular separation between VM- and VL-focused force directions differed significantly from zero (mean ± SD, 34 ± 19°; one-sample *t* test vs 0°, *t*_(7)_ = 5.07; *p* = 0.0014; Cohen’s *d* = 1.79; Wilcoxon signed-rank *p* = 0.0078), indicating that participants reliably altered force direction when switching task focus.

In contrast, the direction of this task-dependent force shift was not consistent across participants. The mean shift-direction vector across subjects was small (mean resultant length *R* = 0.31), and a multivariate Hotelling’s *T*^2^ test revealed no significant deviation from zero (*T*^2^ = 3.13; *F*_(3,5)_ = 0.75; *p* = 0.57). Component-wise one–sample tests on the *x*-, *y*-, and *z*-components were likewise nonsignificant (all *p* > 0.12). Together, these findings indicate that although VM- and VL-focused tasks elicited reliable within-subject changes in force direction, the orientation of these changes varied substantially across individuals, consistent with the use of individualized strategies.

## Discussion

This study investigated whether humans can selectively activate subregions of two quadriceps muscles during low-force isometric contractions. Using intramuscular EMG, HDsEMG, offline decomposition, and real-time feedback, we found that participants could selectively modulate activity in the proximal region of VM but not consistently dissociate distal VM from VL. These findings demonstrate that region-specific modulation within synergistic muscles is possible but only in specific anatomical compartments with explicit feedback.

From the perspective of neural control of movement, these findings indicate that synaptic input to the quadriceps MUs is not limited to a single rigid low-dimensional command shared by all vasti motor neurons. Instead, MU populations within a quadriceps neural module can be biased toward specific compartments when appropriate feedback and task context are provided. This interpretation aligns the concept of MU modes or neural manifolds identified at the population level (Del Vecchio et al., 2023; Hug et al., 2023), suggesting that some of these modes can be differentially expressed on demand. At the same time, the persistent coactivation of VL and distal VM and the rarity of complete dissociation emphasize that voluntary control remains strongly constrained by common synaptic input and by mechanical coupling imposed by the shared tendon.

Although we interpret the observed compartment-specific modulation primarily in terms of differential descending drive to subsets of motor neurons, α-motoneuron output also reflects substantial contributions from peripheral afferent pathways. Modulation of presynaptic inhibition at Ia afferent terminals or task-dependent changes in reflex gain could, in principle, alter the effective synaptic input received by specific MU populations. Spinal presynaptic inhibition mechanisms are dynamically regulated and can be influenced by segmental and descending pathways during voluntary movement and motor learning (Rudomin and Schmidt, 1999).

### Selective modulation and low-dimensional neural control

Motor neurons of synergistic muscles typically receive strong common synaptic input, placing them on a low-dimensional neural manifold that limits independent control (de Luca and Erim, 1994; Farina and Negro, 2015). This is especially true for the quadriceps, where VL and VM often act as a functional unit and exhibit high coherence during natural contractions (Laine et al., 2015). Consistent with this interpretation, selective dissociation of VL and distal VM was rarely achieved in our study, despite explicit instructions to separate their activity.

However, proximal VM consistently showed activation patterns distinct from both distal VM and VL when feedback was provided. During standard ramp contractions without EMG feedback, activity across regions remained highly correlated (>0.8), indicating that this compartment-specific dissociation does not emerge spontaneously but only with targeted feedback. This suggests that neural drive to the quadriceps is more nuanced than whole-muscle analyses typically reveal and supports the existence of latent subregional control that can be accessed under appropriate conditions.

### Reconciling contrasting findings in the literature

Recent work on quadriceps MUs suggests that the CNS operates in a low-dimensional space of common inputs but that access to different dimensions depends on the feedback variable, anatomical sampling, and learning history (Dernoncourt et al., 2025). In addition to voluntary drive and feedback, MU modes can also be altered by changes in afferent input, such as acute muscle stretch (Weinman et al., 2024), indicating that neural modes are state-dependent. This suggests that differences between studies may arise from task context and sensorimotor conditions rather than distinct control strategies.

Recent studies have produced seemingly conflicting results. Del Vecchio et al. (2023) used FA to identify multiple neural modes shared across VL and VM, suggesting variability in the distribution of synaptic inputs to the motor neurons that innervate these muscles. However, these inputs were inferred during steady-state contractions, and volitional access to the underlying modules was never tested. In contrast, Rossato et al. (2024) directly tested volitional dissociation using online HDsEMG decomposition and feedback on individual MU discharge rates but found no dissociation. Several factors reconcile these findings. First, Rossato and colleagues used a feedback variable (individual discharge rates) that is noisy and constrained by common input and intrinsic motor neuron properties, whereas our feedback (RMS intramuscular) provided a smoother, more accessible population-level signal from targeted muscle. Second, selective activation occurred only in proximal VM, an anatomically distinct compartment; distal VM activity resembled VL, consistent with Rossato et al. Third, participants in our study were given a limited exploratory period to discover strategies for biasing the feedback signal, resembling key aspects of operant-conditioning paradigms (Thompson et al., 2009, 2013; Wolpaw, 2010).

Thus, latent partially distinct common synaptic inputs exist, but voluntary access to them requires alignment of the feedback target, muscle compartment, and learning context. The emergence of compartment-specific patterns after a brief exploratory phase with local EMG feedback resembles operant-conditioning paradigms applied to spinal reflexes and cortical activity (Thompson et al., 2009, 2013; Wolpaw, 2010). In this framework, proximal VM represents a latent control dimension normally embedded within a global quadriceps mode but selectively expressed when behavior is shaped by an appropriate feedback rule. This has translational relevance: feedback-guided training could be used to bias neural drive toward specific VM compartments to modulate patellar tracking, redistribute joint loading, or counteract maladaptive coactivation patterns in pathology.

### Anatomical and functional basis of proximal VM selectivity

The proximal VM compartment exhibits distinct architecture and innervation, with more oblique fiber orientation, separate motor branches, and region-specific roles in mediating medial patellar forces (English et al., 1993; Travnik et al., 1995; Gallina et al., 2016; Madarshahian et al., 2021). This anatomical differentiation aligns with our MU decomposition results, where proximal VM showed a larger proportion of uniquely recruited MUs during VM-targeted tasks. In contrast, distal VM units strongly overlapped with VL units, consistent with their shared functional contribution to knee extension torque.

These findings suggest that VM compartmentalization is not merely anatomical but functionally exploitable, at least under explicit feedback. The presence of unique MUs in proximal VM indicates that this compartment receives at least partially independent descending input, which becomes accessible when participants can explore available control strategies.

### Individual variability in motor strategies

MU recruitment patterns and biomechanical strategies were consistent within individuals but differed between individuals. Some participants achieved selective activation through slight internal rotation, whereas others used changes in foot pressure distribution or vertical force adjustments. This variability reflects a key property of the neuromuscular system: individuals discover personalized solutions within the constraints of their anatomy and habitual motor patterns.

This aligns with work showing individual synergies and neuromechanical solutions in other muscle groups (Cakici et al., 2022; Simpetru et al., 2024; Oßwald et al., 2025). It suggests that selective compartmental activation is not a universal control pattern but a learned skill that emerges from self-exploration with appropriate feedback.

### Force direction as a basis for compartmental biasing

Analysis of force vectors applied to the dynamometer revealed that VM- and VL-focused tasks were accompanied by reliable within-subject changes in force direction. The angular separation between VM- and VL-focused contractions was significantly greater than zero, indicating that participants consistently altered the direction of the applied force when switching task focus. However, the orientation of these changes varied across participants, and no common group-level shift in force direction was observed.

This lack of directional consistency indicates that force modulation should not be interpreted as a fixed biomechanical constraint underlying selective compartmental activation. Instead, shifts in force direction likely reflect individualized neuromechanical strategies discovered during the exploratory phase of the task. Participants relied on subtle adjustments in foot pressure, joint loading, or internal torque distribution to modify EMG feedback signal and MU output.

These findings align with the broader observation that selective compartmental activation was achieved through personalized solutions rather than a uniform motor strategy. Shifts in force direction therefore serve as an external signature of internal control strategies rather than as a causal driver of compartment-specific recruitment. This interpretation is consistent with the notion that voluntary access to latent neural dimensions is constrained but flexible, allowing individuals to exploit distinct biomechanical routes within the same task framework.

### Limitations and future directions

Several limitations should be considered. First, selective dissociation of MUs between and within vastii muscles only happened during low-force contractions, limiting generalization to higher-force or dynamic movements. Whether similar compartment-specific control occurs during functional tasks such as walking or stepping remains unknown.

Second, although posture and gross coactivation of remote muscles were visually monitored, EMG was not recorded from muscles other than the quadriceps. Subtle coactivation or biomechanical adjustments may therefore have contributed to some of the individualized strategies. Moreover, MU activity was not characterized by identifying the neural modules (MU modes) associated with VL- and VM-focused tasks (Del Vecchio et al., 2023; Weinman et al., 2024).

Third, a foot support platform facilitated selective activation but was only indirectly coupled to the dynamometer transducer, potentially underestimating absolute vertical force. Accordingly, force analyses were restricted to directional changes and should be interpreted as behavioral correlates of individualized control strategies rather than biomechanical constraints.

Future work should examine whether compartment-specific modulation can be stabilized with extended training, generalized to dynamic tasks, or exploited in rehabilitation contexts where selective activation of VM subregions may be clinically relevant.

### Conclusion

Our study provides evidence that the CNS possesses some capacity to flexibly modulate neural modules within two of the quadriceps muscles. The compartmentalization observed within VM suggests that this muscle is not homogeneously controlled but instead includes regions that can be differentially activated under specific volitional strategies. However, full dissociation between VM and VL or between VM compartments was rare. These findings refine our understanding of neuromuscular control in this large group of synergistic muscles and open new avenues for research and clinical intervention aimed at enhancing selective motor control through feedback-guided training and personalized rehabilitation strategies.

## Data Availability

The datasets generated during the current study are available from the corresponding author upon reasonable request.
